# Clinical validation of a gene expression signature that differentiates benign nevi from malignant melanoma

**DOI:** 10.1111/cup.12475

**Published:** 2015-04-13

**Authors:** Loren E. Clarke, M.B. Warf, Darl D. Flake, Anne‐Renee Hartman, Steven Tahan, Christopher R. Shea, Pedram Gerami, Jane Messina, Scott R. Florell, Richard J. Wenstrup, Kristen Rushton, Kirstin M. Roundy, Colleen Rock, Benjamin Roa, Kathryn A. Kolquist, Alexander Gutin, Steven Billings, Sancy Leachman

**Affiliations:** ^1^ Myriad Genetic Laboratories, Inc. Salt Lake City UT USA; ^2^ Myriad Genetics, Inc. Salt Lake City UT USA; ^3^ Harvard Medical School and Beth Israel Deaconess Medical Center Boston MA USA; ^4^ Department of Dermatology, The University of Chicago Chicago IL USA; ^5^ Department of Pathology Northwestern Memorial Hospital Chicago IL USA; ^6^ Department of Cutaneous Oncology Moffitt Cancer Center Tampa FL USA; ^7^ Department of Dermatology University of Utah Salt Lake City UT USA; ^8^ Cleveland Clinic Cleveland OH USA; ^9^ School of Medicine, Oregon Health & Science University Portland OR USA

**Keywords:** melanoma, molecular diagnositcs, pathology, real time PCR, validation studies

## Abstract

**Background:**

Histopathologic examination is sometimes inadequate for accurate and reproducible diagnosis of certain melanocytic neoplasms. As a result, more sophisticated and objective methods have been sought. The goal of this study was to identify a gene expression signature that reliably differentiated benign and malignant melanocytic lesions and evaluate its potential clinical applicability. Herein, we describe the development of a gene expression signature and its clinical validation using multiple independent cohorts of melanocytic lesions representing a broad spectrum of histopathologic subtypes.

**Methods:**

Using quantitative reverse‐transcription polymerase chain reaction (PCR) on a selected set of 23 differentially expressed genes, and by applying a threshold value and weighting algorithm, we developed a gene expression signature that produced a score that differentiated benign nevi from malignant melanomas.

**Results:**

The gene expression signature classified melanocytic lesions as benign or malignant with a sensitivity of 89% and a specificity of 93% in a training cohort of 464 samples. The signature was validated in an independent clinical cohort of 437 samples, with a sensitivity of 90% and specificity of 91%.

**Conclusions:**

The performance, objectivity, reliability and minimal tissue requirements of this test suggest that it could have clinical application as an adjunct to histopathology in the diagnosis of melanocytic neoplasms.

Skin cancer is the most common cancer worldwide, with melanoma being the most fatal form. Accurate diagnosis of the vast majority of melanocytic lesions can be accomplished with conventional light microscopy by a skilled histopathologist. However, numerous studies have reported inter‐observer variability in some cases, even among experienced dermatopathologists.^1^, ^2^, ^3^, ^4^ Farmer et al. illustrated this discordance with a review of 40 benign and malignant melanocytic lesions; in a panel of eight expert dermatopathologists, diagnostic discordance between two or more panel members was observed in 38% of cases.^1^ Whereas the observed frequency of discordance varies across additional studies, evidence suggests it is a significant problem, even among expert dermatopathologists.

Moreover, diagnosis by expert consensus may not reliably classify some types of melanocytic tumors.^5^, ^6^ In a study by Cerroni et al. experts sought histopathologic criteria that could reliably classify atypical melanocytic tumors as benign or malignant.^7^ Pathologists incorrectly classified 53% of cases with a favorable outcome as malignant and 27% of cases with an unfavorable outcome (local recurrence, metastasis or death) as benign. Similarly, in a recent study of atypical Spitz tumors, 33% of cases with evidence of advanced disease failed to be diagnosed as melanoma by a panel of experts.^8^ Histopathologic assessment may be further limited by the apparent trend toward increasingly smaller biopsies.^9^ Conventional histopathologic criteria that differentiate nevus from melanoma (e.g. symmetry, circumscription, maturation) require evaluation of all or most of the neoplasm. Small samples often contain only part of a lesion and may compel even the best diagnostician to withhold a definitive diagnosis and recommend re‐excision.

Owing to these issues, adjunctive methods have been sought to supplement histopathology for the diagnosis of melanocytic neoplasms. These have included comparative genomic hybridization (CGH), fluorescence *in situ* hybridization (FISH), and microarray technologies.^10^, ^11^, ^12^, ^13^, ^14^, ^15^, ^16^, ^17^ Each has shown utility in some situations but limitations still exist.^18^, ^19^ We sought to determine if quantitative reverse transcription PCR (qRT‐PCR) could be used to identify groups of genes that are differentially expressed between malignant and benign melanocytic lesions in a manner that could be clinically applicable. We sought solely to explore the gene expression differences between benign nevi and primary melanoma samples (not examining the differences between benign nevi, primary melanomas and metastatic melanomas), as this differentiation would be of the highest clinical value.

With a training cohort of 464 melanocytic lesions, we developed a 23‐gene expression signature that effectively differentiated benign and malignant melanocytic neoplasms. We then assessed performance of the signature in an independent cohort of 437 malignant melanomas and benign nevi.

## Materials and methods

### Sample cohorts

All testing was performed on archival formalin‐fixed paraffin‐embedded (FFPE) tissue sections of melanocytic lesions. Specimens were selected by experienced dermatopathologists and represented a broad spectrum of clinical and histopathologic subtypes, including banal/common nevi (junctional, compound, and intradermal), junctional and compound dysplastic nevi, Spitz nevi, Reed nevi, blue nevi and other subtypes. Non‐melanocytic lesions, metastatic melanomas and melanocytic lesions not primary to skin were excluded. Re‐excision specimens were excluded but melanocytic neoplasms that were inflamed and/or traumatized were included. The training cohort initially contained 595 melanocytic lesions acquired from The University of Munich and Provitro (Berlin, Germany). The validation cohort initially consisted of 571 melanocytic lesions acquired from The Cleveland Clinic, University of South Florida, Northwestern University and the University of Utah.

Each case underwent review by a second expert dermatopathologist (S.T.) who was blinded to the diagnosis of the contributing dermatopathologist. If there was discordance between the diagnoses, the case was reviewed in a blinded manner by a third expert dermatopathologist (C.S.) for adjudication. Regardless of their preferred terminology for various subtypes, the reviewing dermatopathologists were encouraged to designate each case as either benign or malignant (rather than ‘indeterminate’ or ‘of uncertain malignant potential’) such that difficult cases would not be excluded from the cohorts. Diagnostic concordance was maximized because the contributing dermatopathologists provided lesions for which they felt a definitive diagnosis could be established. This study was conducted with institutional (institutional review board) oversight.

### Measurement of gene expression

Archival FFPE melanocytic lesions were used for these studies. Each case required one H&E stained slide and 2–5 slides containing a total of 20 µm of unstained tissue, with a minimal requirement of 0.5 mm by 0.1 mm of melanocytic tissue. An area representative of the lesions was identified and circled on the H&E slide by an anatomic pathologist, and the corresponding area was macro‐dissected from the unstained tissue slides and pooled into a single tube. RNA was extracted from the tissue using the RNeasy FFPE kit (Qiagen, Valencia, CA, USA) [run on a QIACube instrument (Qiagen), then nuclease treated using DNAse I (Sigma‐Aldrich, St. Louis, MO, USA), and quantified on a Nanodrop spectrophotometer (Thermo Scientific, Waltham, MA, USA)]. RNA samples were normalized to 40 ng/µl, or were tested at the current concentration if <40 ng/µl.

RNA was next used for cDNA synthesis using the High Capacity cDNA Reverse Transcription Kit (Life Technologies, Carlsbad, CA, USA). Genes of interest were pre‐amplified for 14 cycles, according to the manufacturer's guidelines (Life Technologies) using the TaqMan PreAmp Master Mix and an associated TaqMan amplicon for each target gene [Note: the specific sequence of these primers are proprietary (Life Technologies)]. Finally, TaqMan quantitative PCR was used to measure the expression of each gene using the TaqMan Universal PCR Master Mix and custom TaqMan Low Density Array (TLDA) cards, analyzed on an Applied Biosystems 7900HT instrument.

All samples were run in triplicate by dividing each sample into three aliquots after cDNA synthesis, but prior to preamplification. Expression levels for each gene were calculated using the standard ΔΔ*C*_T_ method.^20^ In brief, the expression values of each gene were measured by determining the *C*_T_ (crossing threshold) of each gene. The *C*_T_ of each gene for each sample replicate was normalized by the average *C*_T_ of housekeeper genes on the same replicate, yielding Δ*C*_T_. The ΔΔ*C*_T_ was calculated by centering the Δ*C*_T_ values of each gene by the average of the Δ*C*_T_ values of the gene across all samples in the training cohort. The median replicate ΔΔ*C*_T_ value was used for each amplicon. Two of the three replicates of each gene on each sample were required to be within two ΔΔ*C*_T_ units of each other to be considered appropriately measured.

### Development of the gene expression signature

Genes with highly correlated expression and similar biological function were averaged into a single measurement to differentiate benign and malignant melanocytic tumors (see Supporting Information for more information). The selection of these genes will be addressed in more detail the Results section. Consolidated gene groups and single genes were subjected to forward selection in a series of logistic regression models. The p‐value from a likelihood ratio test comparing the models with and without each new variable was used as the criterion for inclusion. Variables were sequentially selected by their ability to enhance the discriminatory power of the previously selected variables, until the addition did not result in significant improvement (Bonferroni adjusted p‐value <0.05).

The logistic regression model resulting from the final selection of genes was refined to allow for a more realistic fit of the model to the data. Specifically, parameters were added to estimate the false negative and the false positive rates of the best set of genes. Maximum likelihood estimation was used to fit this non‐standard model. A single melanoma diagnostic score (melanoma diagnostic score) was produced by applying the optimal linear combination of the individual components' expression, as derived in the generalized logistic regression model. A melanoma diagnostic score of zero (inflection point of the probability curve) was chosen as the threshold to differentiate melanocytic nevi and melanoma. The signature was further refined to improve its technical robustness by supplementing the measurements of the components in the model that were represented by one measurement (see Supporting Information for more detail). A final TLDA card with all the selected amplicons was designed and the component weights were adjusted to make the score from the final TLDA card (after the refinements) match the score from the original TLDA card (prior to the refinements) so that the components with supplemental measurements were not over‐weighted.

### Validation of the gene expression signature

Samples from the validation cohort were used to validate the pre‐defined gene expression signature with a pre‐defined cutoff of zero to differentiate melanoma and nevi. The association between the melanoma diagnostic score and pathologic diagnosis was assessed using the Wilcoxon rank‐sum test. Exact 95% confidence intervals (CIs) were computed for sensitivity and specificity based on the binomial distribution. The melanoma diagnostic score was then used to assess the performance of the gene expression signature within specific histopathologic subtypes. Data analysis was only performed on subtypes with ≥30 samples to avoid issues with sampling artifacts.

## Results

### Development of a multivariate gene expression signature

We first identified 79 candidate biomarker genes whose expression might prove useful in a gene signature to differentiate benign nevi and malignant melanoma. These biomarkers were selected because they have been previously observed in literature to have differential expression in benign nevi and malignant melanoma, or were internally observed to have increased expression in aggressive tumors. The RNA expression of these 79 biomarkers was initially evaluated in a dataset of 83 melanocytic lesions, and the 79 markers were narrowed to a smaller set of the 40 most promising biomarkers that could be evaluated in greater detail. See Table S1 for additional details on the selection of candidate biomarkers and their initial evaluation.

We then evaluated the RNA expression of a refined set of the 40 most promising candidate genes in a large training cohort of 595 melanoma and nevus samples (see Supporting Information for details on the selection of these 40 genes). In the initial cohort of 595 melanocytic lesions, 51 samples were excluded due to re‐excision, lack of sufficient tissue for processing, or because classification as benign or malignant could not be made definitively. Of the remaining 544 samples, 42 were excluded because we could not reliably measure the housekeeping genes' expression. Additionally, expression for one or more genes of interest could not be detected in 38 samples. Thus, 464 samples produced sufficient data for analysis. The histopathology of the samples used in this study are presented in Table 1.

**Table 1:** Distribution of melanocytic lesions by subtype

Melanocytic lesions	Cohort		Total
	Training	Validation	
*Melanomas*			
Superficial spreading	167	105	**272**
Nodular	23	38	**61**
Acral	20	9	**29**
Lentigo maligna/Lentigo maligna melanoma	39	31	**70**
Other	5	28	**33**
**Total**	**254**	**211**	**465**
*Nevi* *			
Compound	68	101	**169**
Junctional	38	20	**58**
Intradermal	28	41	**69**
Spitz	34	7	**41**
Blue	38	22	**60**
Other	4	35	**39**
**Total**	**210**	**226**	**436**

* Includes dysplastic nevi [n = 117 (Training) and n = 67 (Validation)].

In the 464 sample training cohort, 27 of the 40 candidate genes were able to differentiate the melanoma and nevi samples with an area under the curve (AUC) that was >70% (Fig. S1). To build a diagnostic signature from this dataset, we first determined which genes had correlated expression and could be assessed as a single averaged component (see Supporting Information for more information on this analysis). In brief, genes with both highly correlated expression and similar biological functions were assessed as a single averaged component. All 10 cell cycle progression genes were averaged into one component (Table S1), and eight genes within the immune cluster were averaged in a second component (*CCL5*, *CD38*, *CXCL10*, *CXCL9*, *IRF1*, *LCP2*, *PTPRC* and *SELL*). All the remaining genes were assessed on an individual basis.

**Figure 1:**
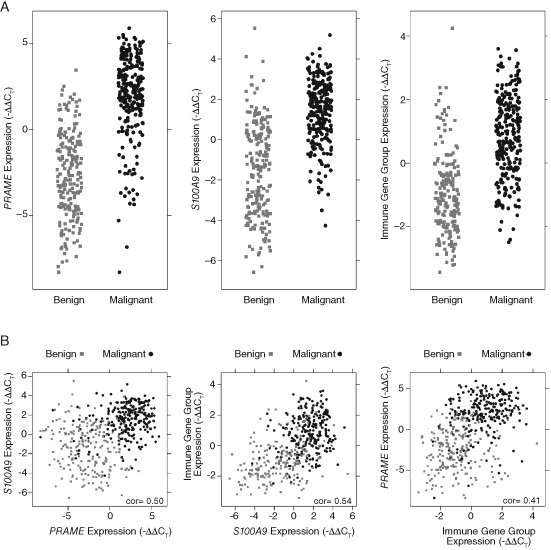
Distribution of the gene expression for the three components in the best performing multivariate signature. A) The individual distribution of RNA expression in benign (grey) and malignant (black) samples in the training cohort for each of the three components. B) Comparison of the RNA expression of each component to each of the other two components in the training cohort. The low correlation (cor) of each comparison is noted.

We next used forward selection and traditional logistic regression to explore the performance of various diagnostic models (preliminary signature) that included different combinations of the individual genes and two gene components. *PRAME* expression was the single most effective differentiating feature (*p* = 1.2 × 10^−68^) and was the first gene added to the preliminary signature. Through forward selection, the preliminary signature was improved first by the addition of *S100A9* and was further improved by the addition of the eight gene immune component (*PRAME*: *p* = 4.5 × 10^−68^ *S100A9*, *p* = 3.9 × 10^−12^ immune average, *p* = 7.2 × 10^−5^) (Fig. 1A). The diagnostic power of the preliminary signature was not improved by incorporating any additional genes, or by adding the cell cycle progression gene component (Table S1).

Thus, the optimal diagnostic gene signature consisted of three components: PRAME, S100A9 and the eight gene immune component (CCL5, CD38, CXCL9, CXCL10, IRF1, LCP2, PTPRC and SELL). Each component within this signature had a distinct expression profile and was not highly correlated with the expression of the other components (Fig. 1B), verifying that each component contributes independent information to the signature. The three‐component signature was further refined using generalized logistic regression, which significantly improved the fit (likelihood ratio test p = 6.0 × 10^−4^). This refined multivariate signature generated a melanoma diagnostic score for each sample from the 464 sample training cohort, with a resulting AUC of 95%. Setting a score of zero as the threshold to differentiate nevi and melanoma, the sensitivity of the signature was determined to be 89% and the specificity to be 93% (Fig. S3; Table 2).

**Table 2:** List of genes included in the final multivariate signature

Genes		
Component 1	Component 2	Component 3‡
PRAME *	S100A9	CCL5
	S100A7 †	CD38
	S100A8 †	CXCL10
	S100A12 †	CXCL9
	PI3 †	IRF1
		LCP2
		PTPRC
		SELL

Housekeeping genes included: CLTC, MRFAP1, PPP2CA, PSMA1, RPL13A, RPL8, RPS29, SLC25A3 and TXNL1.

* PRAME gene expression represents the average of two amplicon measurements.

† These genes were added to the gene expression signature after evaluation of the signature with the training cohort.

‡ These eight immune genes were evaluated as an averaged group in the multivariate signature.

To improve the technical robustness of the signature for a production setting, additional measurements were added to three different components (see the Supporting Information for additional detail). First, we added another amplicon to measure the expression of PRAME, with the PRAME component of the melanoma diagnostic score being the average of the two measurements. Next, four genes with highly correlated expression (S100A7, S100A8, S100A12 and PI3) were added to the S100A9 component, with the resulting S100A9 component of the melanoma diagnostic score being the averaged expression of all five genes (see Supporting Information). Finally, we also increased the number of housekeeper genes from five to nine. In a 77 sample concordance study, we verified that these additions did not alter the performance of the gene expression signature. Thus, the refined gene signature had a total of 24 measurements of 23 different genes.

### Validation of the gene expression signature

The signature was validated in a cohort of 571 samples, derived from four different institutions that were independent from the contributing institutions in the training cohort. A melanoma diagnostic score was determined for these samples using the pre‐determined score calculation that was developed from the training cohort, with a pre‐defined cutoff of zero to differentiate melanoma and nevi.

Of the initial cohort of 571 melanocytic lesions, 20 samples were excluded because they were determined to be re‐excision specimens. Another 24 were excluded based on insufficient tissue for processing or lack of a consensus diagnosis. Ninety samples did not produce a score based on gene signature expression levels being below the detection threshold. Thus, the final validation cohort was comprised of 437 samples, which represented several melanoma subtypes (n = 211), as well as both dysplastic (n = 67) and conventional nevi (n = 149). The histopathology of all samples in the validation cohort is presented in Table 1. (The mean and median Breslow depths of the 211 melanoma samples were 1.74 and 0.82 mm, respectively, with a range of 0.10–29.0 mm).

**Figure 2:**
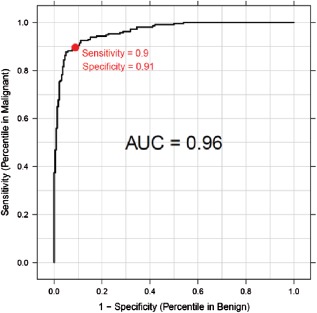
Receiver operating characteristic (ROC) curve of diagnostic scores in the clinical validation cohort.

**Figure 3:**
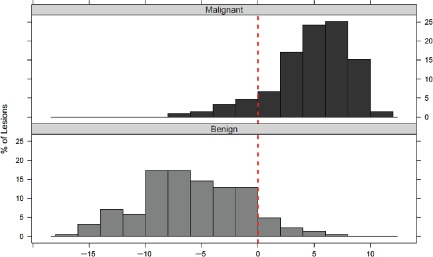
Distribution of diagnostic scores in the clinical validation cohort.

The signature performance was determined in the cohort of 437 samples by comparing the melanoma diagnostic score to the consensus histopathologic diagnosis. Using the pre‐defined cutoff score of zero established in the training cohort, the sensitivity and specificity were determined to be 90% (95% CI: 85–93%) and 91% (95% CI: 87–95%), respectively (Fig. 2). The range of scores from the validation cohort followed a bimodal distribution, with the majority of malignant lesions producing a score greater than zero and the benign lesions a score less than zero (Fig. 3). The scores ranged from −16.7 to +11.1. The AUC value was 96% (p = 3.7 × 10^−63^).

During the histopathologic review, adjudication review was necessary for 17 of the 437 validation samples, indicating that these 17 cases were histopathologically ambiguous. Nine of these cases had an adjudicated diagnosis of malignant, all of which were classified as malignant by the signature. Of the eight cases for which the adjudicated diagnosis was benign, the signature classified four as benign and four as malignant.

Performance of the gene signature was also assessed within specific histopathologic subtypes (Table 3). The specificity in compound and dermal nevi were each similar to the specificity within all nevi. The sensitivity within each melanoma subtype was also similar to the overall sensitivity for all melanoma samples. Thus, the performance of the gene signature within these histopathologic subtypes was similar to the overall performance of the gene signature.

**Table 3:** Performance of the signature within individual subtypes*

Pathologist classification	Signature classification		Signature performance	
	Malignant	Benign	Sensitivity	Specificity
All melanomas			90%	
Superficial spreading	90	15	86%	
Nodular	37	1	97%	
Lentigo maligna	28	3	90%	
All nevi†				**91%**
Compound	6	95		94%
Intradermal	1	40		98%

* Results reported only for subtypes with ≥30 samples.

† Compound nevi group contained 52 dysplastic nevi.

The performance of a signature generally decreases upon validation because of over‐fitting cohort specific differences between case and control samples in the training dataset. In this study, the performance of the signature was similar in both the training and validation cohorts, indicating that the signature was not significantly over‐fit in its development. To confirm this observation, the same generalized logistic regression used in training was used to determine the optimal fit of the signature within the validation cohort. The melanoma diagnostic score and the optimal score were nearly identical, with a correlation of 99%.

## Discussion

In this study of melanocytic tumors, the melanoma diagnostic score differentiated conventional melanocytic nevi from melanoma with a sensitivity of 90% and a specificity of 91%. Further validation is necessary, but based upon the results in this retrospective cohort, the signature appears applicable to a broad array of melanocytic tumors, including some that might prove challenging to classify by histopathology alone. This is likely due, in part, to the fact that the original training cohort included a broad spectrum of melanocytic lesions, including lesions that were somewhat ambiguous. However, the requirement for diagnostic concordance among two expert dermatopathologists helped to ensure that diagnoses for the studied lesions were accurate.

Importantly, the score established using the training cohort was evaluated in an entirely separate validation cohort. Similar to the training cohort, this validation cohort also included a broad spectrum of histopathologic subtypes. Some were ‘classic’ examples of various nevus and melanoma subtypes, but lesions from subtypes known to be associated with diagnostic uncertainty were also included. Subset analyses of results by nevus and melanoma subtypes indicated that the signature performed with 94–98% specificity and 86–97% sensitivity. Whereas the total number of samples analyzed in common subtypes is large, additional studies will be required to validate the signature's performance in uncommon nevus and melanoma subtypes. Additionally, it should be noted that metastatic lesions were purposefully excluded in these studies. Gene expression patterns within a primary melanoma can change drastically upon metastasis, causing a large number of genes (including some within this signature) to change their expression.^11^, ^13^, ^14^, ^15^ Thus, this gene expression signature should only be used in the context of differentiating benign nevi and primary melanomas. However, there is significant clinical value in having an adjunctive tool for diagnosing primary melanomas, not metastatic melanomas.

The relatively high sensitivity and specificity are encouraging, but given the clinical consequences of misclassifying a malignant melanoma as a benign nevus, it is important for the sensitivity to be maximized. To this end, an indeterminate zone could be introduced. Approximately half of the malignant lesions (5%) that appear to have been misclassified by the signature in the validation cohort have a score between −2 and 0. Excluding all scores in this range from the clinical validation cohort would bring the signature sensitivity to 94% and a specificity of 90%.

This signature differs from other widely used molecular diagnostic methods for melanoma in that the other assays rely upon identification of chromosomal abnormalities in melanocytes.^21^, ^22^, ^23^ Analysis by qRT‐PCR can detect changes in gene expression that may not result from gains or losses of DNA. This gene expression signature also contributes additional molecular information by assessing the gene expression of *PRAME*, a known melanoma tumor antigen. For example, *PRAME* expression is strongly reduced in the melanocytes of benign nevi,^11^ but significantly higher in 88% of primary melanomas and 95% of metastatic melanomas.^24^ However, its expression appears to result from changes in the methylation status rather than chromosomal gains or deletions.^24^

In addition, it is becoming clear that the malignant potential of melanoma is at least partially determined by the interaction of the tumor with its microenvironment. The fact that both *S100A9* and the other immune signaling gene group are critical components of the signature appears to reflect the importance of this interaction. Certain *S100* protein subtypes, particularly *S100A8* and *S100A9*, play a role in the inflammatory response to many types of neoplasms.^25^ Melanomas expressing *CCL5*, *CXCL9* and *CXCL10* have been associated with prolonged patient survival and are more likely to respond to ipilimumab, a human monoclonal antibody that improves overall survival in metastatic melanoma patients.^26^, ^27^

One potential limitation of this study is that it was carried out with archived FFPE tissue. mRNA extracted from archived FFPE samples is more prone to fragmentation when compared with recently prepared FFPE lesions. This might explain the relatively high sample failure rate of the signature in older samples. Indeed, we observed a much lower failure rate in contemporary samples (data not shown). Samples with low scores have an increased chance of failure if the mRNA is from an older sample and is degraded, which could bias estimates of the sensitivity and specificity. To mitigate this bias, both the training and validation cohorts consisted of a variety of older archival samples and newer contemporary samples.

Given the prevalence of melanocytic neoplasms that are ambiguous or difficult to classify by histopathology alone, there is a great need for adjunctive tests that could enhance reproducibility and diagnostic accuracy. The development and validation of the gene expression signature presented here consists of a total of 901 samples. The strong agreement between the performance of both the training and validation cohorts suggests that this method accurately assesses the performance of the gene expression signature to differentiate malignant melanoma and benign nevi. Additional outcomes‐based and prospective studies are needed to further assess the performance of this test.

## Supporting information


**Appendix S1.** Supplemental methods and results.

**Fig. S1.** Performance of the 40 genes tested in the training cohort. The performance of each gene is measured by the area under the curve (AUC) of each gene when differentiating benign and malignant melanocytic lesions.

**Fig. S2.** Clustering of the gene expression of the 40 genes evaluated in the training cohort. The three main clusters are annotated. CCP, cell cycle progression.

**Fig. S3.** Performance of the best multivariate model in the training cohort. A) Distribution of the diagnostic score from the model in malignant and benign samples. B) The receiver operating characteristic (ROC) curve of the model, with the sensitivity and specificity displayed at the chosen cutoff point. The area under the curve (AUC) of the ROC is also noted.

**Table S1.** List of the 79 candidate biomarker genes.

**Table S2.** Performance of the candidate diagnostic genes in a multivariate model using the training cohort.
