# Supplementary material for: Clinical validation of a gene expression signature that differentiates benign nevi from malignant melanoma
Source: J Cutan Pathol. 2015 Apr 13;42(4):244–52. doi: 10.1111/cup.12475 (PMC6681167; doi:10.1111/cup.12475)
Supplement: Supplementary file 1 — Appendix S1. Supplemental methods and results. [file CUP-42-244-s005.doc]

**Clinical Validation of a Gene Expression Signature that Differentiates Benign Nevi from Malignant Melanoma**

**Supplemental Methods and Results**

**Selection and Initial Evaluation of 79 Candidate Biomarker Genes**

We identified and evaluated 79 candidate genes whose RNA expression had the potential to differentiate benign and malignant melanocytic tumors (Supplemental Table 1). These genes were selected because they have been observed to have differential expression in benign nevi and malignant melanoma1-9 or showed increased expression in more aggressive cancers. This list includes genes with known immune functions, cell cycle progression (CCP) genes, as well as genes that regulate cellular differentiation, Notch signaling, migration, fat metabolism and the cytoskeleton.

We evaluated the RNA expression of these 79 biomarkers in 83 melanocytic lesions (52 benign and 31 malignant lesions) using quantitative RT-PCR (see “Quantification of Gene Expression” in Methods). Some of the genes were able to differentiate between the melanoma and nevus samples in a highly significant manner. Genes were ranked for individual performance to facilitate selection of genes for the next round of studies. By using a conservative area under the curve (AUC) cutoff of 70%, we chose 40 genes for further assessment (Supplemental Table 2). All CCP genes were similarly effective when differentiating benign and malignant melanocytic lesions (AUC >70%) and had highly correlated expressions. Thus, only the 10 most effective CCP genes were evaluated.1, 9 The 30 most effective non-CCP genes with an AUC >70% were also chosen for additional evaluation. All genes with an AUC <70% or with insufficient technical performance (missing expression values in a large number of samples) were excluded from further study.

**Cluster Analysis and Consolidation of Correlated Genes**

Since many of the 40 genes evaluated in the training cohort are involved in similar biological pathways, we determined which of the 40 genes had correlated expression across the 544 training cohort by performing a clustering analysis. Genes with both highly correlated gene expression and similar biological functions were then consolidated into averaged components for assessment during the development of the gene expression signature.

To perform the cluster analysis, one minus the absolute value of the pair-wise Spearman correlation between genes was used as the “distance” metric and Ward’s method was used as the criterion to form the clusters. We observed two different groups of strongly correlated genes, with the remaining genes forming a third loose cluster (Supplemental Figure 2).

The *S100A9* and all 10 CCP genes formed one cluster. This was expected as these CCP genes have been observed to have highly correlated expression in other cancer. 1, 9, 10 It was not expected for the CCP genes to cluster with *S100A9*, which is a multifunctional protein that is thought to be involved in various immune pathways. However, the average correlation of *S100A9* with each of the CCP genes was less than the average pairwise correlation between CCP genes, and the range in expression of *S100A9* was much greater than any of the CCP genes. Thus, due to functional differences and a lower degree of correlation with the CCP genes, *S100A9* was further evaluated on an individual basis. In contrast, all 10 CCP gene expression values were averaged into a single component for further analysis, as we have also done in previous studies.1, 9, 10

The second main cluster consisted of 12 genes with known immune functions. This subset of genes could represent a specific immune pathway (as opposed to a generic immune signature), as only 12 of the 23 genes with known immune functions are clustered within this group. Similar to the CCP genes, 10 of the 12 immune genes had highly correlated expression across the 544 samples, and we excluded these two genes that were not highly correlated (*HCLS1* and *PECAM1*) from an averaged gene group. The *PTPN22* and *CXCL13* genes were also excluded from an averaged group, due to insufficient technical performance (i.e. too many samples missing values, due to low expression and/or technical failures). Thus, we created an averaged group of eight immune genes for further analysis, consisting of the genes *CCL5*, *CD38*, *CXCL9*, *CXCL10*, *IRF1*, *LCP2*, *PTPRC* and *SELL*. The four immune genes excluded from this averaged set were evaluated on an individual basis.

The remaining 17 genes were within a loose “miscellaneous” cluster that consisted of genes with a variety of known functions, such as immune response, signaling, structural regulation, fat metabolism and cell differentiation. As this group was only loosely clustered and did not have functional similarities, each gene within the cluster was evaluated on an individual basis.

**Gene Signature Refinement**

The benefit of the eight gene immune component is that eight averaged measurements improve the technical reproducibility of that component. We wanted to also improve the technical robustness of the *PRAME* and *S100A9* components by similarly having multiple measurements for each gene. To this end, we added a second TaqMan amplicon to measure *PRAME*, with the *PRAME* measurement being an average of the two amplicons. Unfortunately, no other TaqMan amplicons were available for *S100A9*, so we added measurements of four genes that were highly correlated with *S100A9*, which were *S100A7*, *S100A8*, *S100A12* and *PI3* (which were not initially evaluated in these studies). This created an averaged five gene *S100A9*-related component. To improve the technical robustness of the normalization genes, we also increased the number of housekeeper genes from five to nine, using additional housekeeper genes that are well characterized in previous studies. 1, 9, 10

We wanted to verify that these refinements did not alter the performance of the signature, so a concordance study was performed on 77 samples. We compared the data generated with and without the technical refinements and observed a 99% concordance when comparing samples across the two datasets. This indicates the technical improvements do not alter the performance of the gene signature.

Thus, the refined gene signature had a total of 24 measurements, from 23 different genes: *PRAME* (two averaged measurements), an averaged group of five *S100A9*-related genes (*S100A7*, *S100A8*, *S100A9*, *S100A12* and *PI3*), an averaged group of eight immune genes (*CCL5*, *CD38*, *CXCL10*, *CXCL9*, *IRF1*, *LCP2*, *PTPRC* and *SELL*), and nine averaged housekeeper genes used for normalization.

**Supplemental Table 1. List of the 79 candidate biomarker genes**

| *ARPC2* | *CXCL12* | *IRF1* | *PRC1* |
| --- | --- | --- | --- |
| *ASF1B* | *CXCL13* | *IRF4* | *PTN* |
| *ASPM* | *CXCL9* | *ITGB2* | *PTPN22* |
| *BCL2A1* | *DLGAP5* | *KIAA0101* | *PTPRC* |
| *BIRC5* | *DTL* | *KIF11* | *PTTG1* |
| *BUB1B* | *FABP7* | *KIF20A* | *RAD51* |
| *CCL19* | *FN1* | *KRT15* | *RAD54L* |
| *CCL3* | *FOXM1* | *LCP2* | *RGS1* |
| *CCL5* | *GDF15* | *MCM10* | *RRM2* |
| *CD38* | *HCLS1* | *NCOA3* | *S100A9* |
| *CDC20* | *HEY1* | *NR4A1* | *SELL* |
| *CDCA3* | *HLA-DMA* | *NUSAP1* | *SERPPINB4* |
| *CDCA8* | *HLA-DPA1* | *ORC6L* | *SKA1* |
| *CDK1* | *HLA-DPB1* | *PBK* | *SOCS3* |
| *CDKN3* | *HLA-DRA* | *PECAM1* | *SPP1* |
| *CENPF* | *HLA-E* | *PHACTR1* | *TK1* |
| *CENPM* | *IFI6* | *PHIP* | *TOP2A* |
| *CEP55* | *IGHM* | *PLK1* | *WIF1* |
| *CFH* | *IGJ* | *POU5F1* | *WNT2* |
| *CXCL10* | *IGLL5;CKAP2* | *PRAME* |  |

**Supplemental Table 2. Performance of the candidate diagnostic genes in a multivariate model using the training cohort**.

| **Gene** | **Function** | ***p*-value in multivariate model** |
| --- | --- | --- |
| *CENPF* | Cell Cycle Progression | NS* |
| *CEP55* | Cell Cycle Progression | NS* |
| *DLGAP5* | Cell Cycle Progression | NS* |
| *DTL* | Cell Cycle Progression | NS* |
| *FOXM1* | Cell Cycle Progression | NS* |
| *MCM10* | Cell Cycle Progression | NS* |
| *PBK* | Cell Cycle Progression | NS* |
| *PLK1* | Cell Cycle Progression | NS* |
| *RRM2* | Cell Cycle Progression | NS* |
| *SKA1* | Cell Cycle Progression | NS* |
| *PRAME* | Cell Differentiation | 4.5 x 10^-28^ |
| *PTN* | Cell Differentiation | NS |
| *FABP7* | Fat Metabolism | NS |
| *S100A9* | Immune | 3.9 x 10^-12^ |
| *CCL5* | Immune | 7.2 x 10^-5 †^ |
| *CD38* | Immune | 7.2 x 10^-5 †^ |
| *CXCL10* | Immune | 7.2 x 10^-5 †^ |
| *CXCL9* | Immune | 7.2 x 10^-5 †^ |
| *IRF1* | Immune | 7.2 x 10^-5 †^ |
| *LCP2* | Immune | 7.2 x 10^-5 †^ |
| *PTPRC* | Immune | 7.2 x 10^-5 †^ |
| *SELL* | Immune | 7.2 x 10^-5 †^ |
| *BCL2A1* | Immune | NS |
| *CCL3* | Immune | NS |
| *CFH* | Immune | NS |
| *CXCL13* | Immune | NS |
| *HCLS1* | Immune | NS |
| *HLA-DMA* | Immune | NS |
| *HLA-DRA* | Immune | NS |
| *IFI6* | Immune | NS |
| *IGJ* | Immune | NS |
| *ITGB2* | Immune | NS |
| *PECAM1* | Immune | NS |
| *PTPN22* | Immune | NS |
| *RGS1* | Immune | NS |
| *SPP1* | Immune | NS |
| *FN1* | Signaling | NS |
| *HEY1* | Signaling | NS |
| *KRT15* | Structural | NS |
| *PHACTR1* | Structural | NS |

*p-*values are Bonferonni adjusted.

NS, not significant (*p*-value >0.05).

* All 10 cell cycle progression genes were evaluated as an averaged group in the multivariate model.

† These eight immune genes were evaluated as averaged group in the multivariate model.

**Supplemental Figure 1. Performance of the 40 genes tested in the training cohort.** The performance of each gene is measured by the AUC of each gene when differentiating benign and malignant melanocytic lesions.


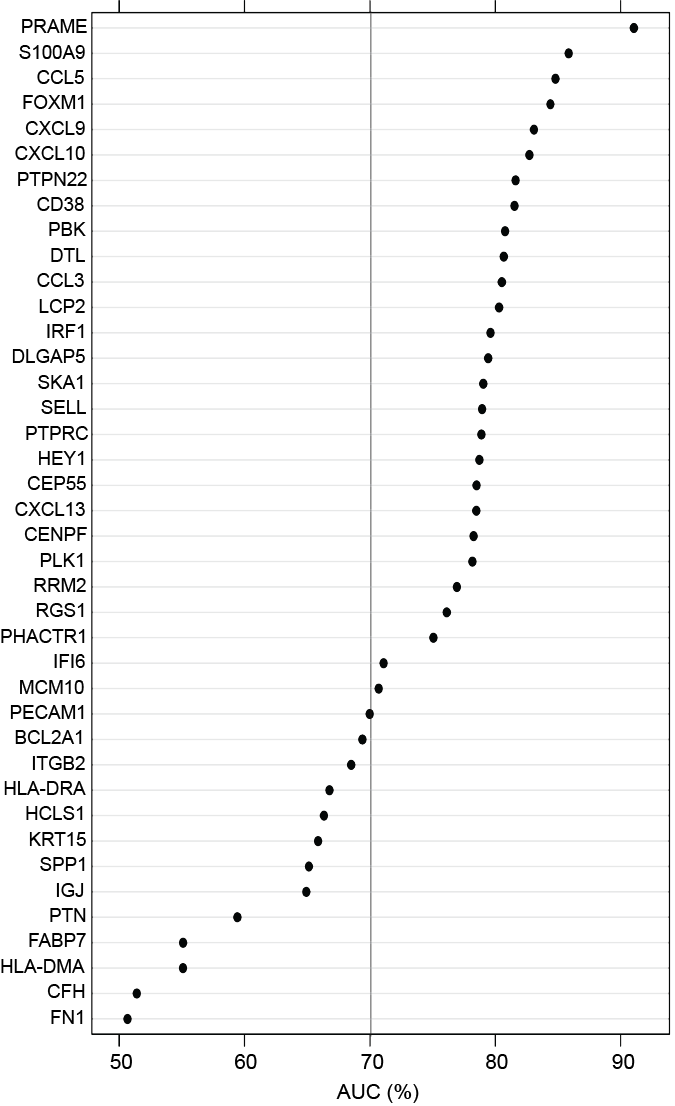


**Supplemental Figure 2. Clustering of the gene expression of the 40 genes evaluated in the training cohort.** The three main clusters are annotated. CCP=cell cycle progression.


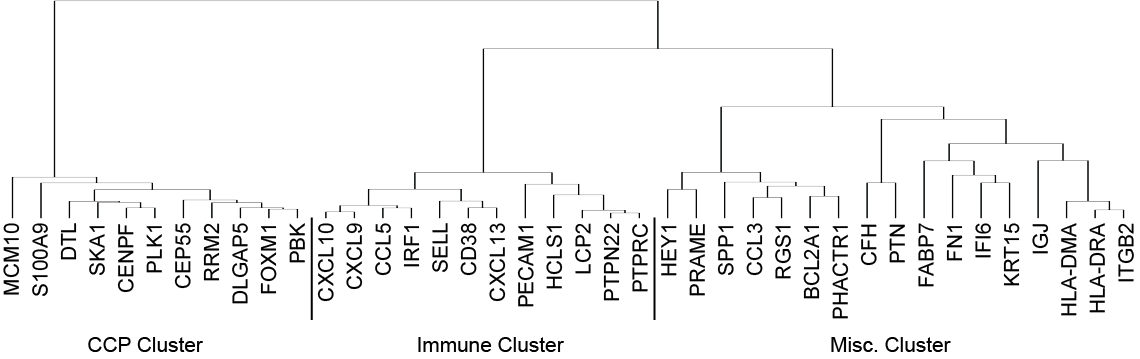


**Supplemental Figure 3. Performance of the best multivariate model in the training cohort.** A) Distribution of the diagnostic score from the model in malignant and benign samples. B) The ROC curve of the model, with the sensitivity and specificity displayed at the chosen cutoff point. The AUC of the ROC is also noted.


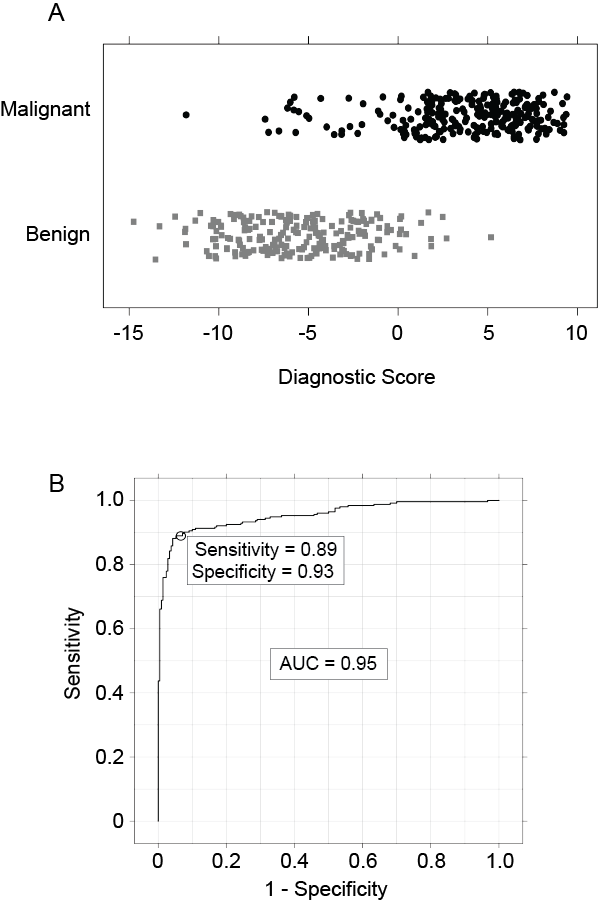


**REFERENCES**

1. Cuzick J, Swanson GP, Fisher G, Brothman AR, Berney DM, Reid JE et al. Prognostic value of an RNA expression signature derived from cell cycle proliferation genes in patients with prostate cancer: a retrospective study. Lancet Oncol 2011;12:245-55.

2. Haqq C, Nosrati M, Sudilovsky D, Crothers J, Khodabakhsh D, Pulliam BL et al. The gene expression signatures of melanoma progression. Proc Natl Acad Sci U S A 2005;102:6092-7.

3. Koh SS, Opel ML, Wei JP, Yau K, Shah R, Gorre ME et al. Molecular classification of melanomas and nevi using gene expression microarray signatures and formalin-fixed and paraffin-embedded tissue. Mod Pathol 2009;22:538-46.

4. Mauerer A, Roesch A, Hafner C, Stempfl T, Wild P, Meyer S et al. Identification of new genes associated with melanoma. Exp Dermatol 2011;20:502-7.

5. Scatolini M, Grand MM, Grosso E, Venesio T, Pisacane A, Balsamo A et al. Altered molecular pathways in melanocytic lesions. Int J Cancer 2010;126:1869-81.

6. Smith AP, Hoek K , Becker D. Whole-genome expression profiling of the melanoma progression pathway reveals marked molecular differences between nevi/melanoma in situ and advanced-stage melanomas. Cancer Biol Ther 2005;4:1018-29.

7. Talantov D, Mazumder A, Yu JX, Briggs T, Jiang Y, Backus J et al. Novel genes associated with malignant melanoma but not benign melanocytic lesions. Clin Cancer Res 2005;11:7234-42.

8. Wachsman W, Morhenn V, Palmer T, Walls L, Hata T, Zalla J et al. Noninvasive genomic detection of melanoma. Br J Dermatol 2011;164:797-806.

9. Wistuba, II, Behrens C, Lombardi F, Wagner S, Fujimoto J, Raso MG et al. Validation of a proliferation-based expression signature as prognostic marker in early stage lung adenocarcinoma. Clin Cancer Res 2013;19:6261-71.

10. Cuzick J, Berney DM, Fisher G, Mesher D, Moller H, Reid JE et al. Prognostic value of a cell cycle progression signature for prostate cancer death in a conservatively managed needle biopsy cohort. Br J Cancer 2012;106:1095-9.
