# Supplementary material for: Clinical validation of a gene expression signature that differentiates benign nevi from malignant melanoma
Source: J Cutan Pathol. 2015 Apr 13;42(4):244–52. doi: 10.1111/cup.12475 (PMC6681167; doi:10.1111/cup.12475)

**Supplemental Figure 1. Performance of the 40 genes tested in the training cohort.** The performance of each gene is measured by the AUC of each gene when differentiating benign and malignant melanocytic lesions.


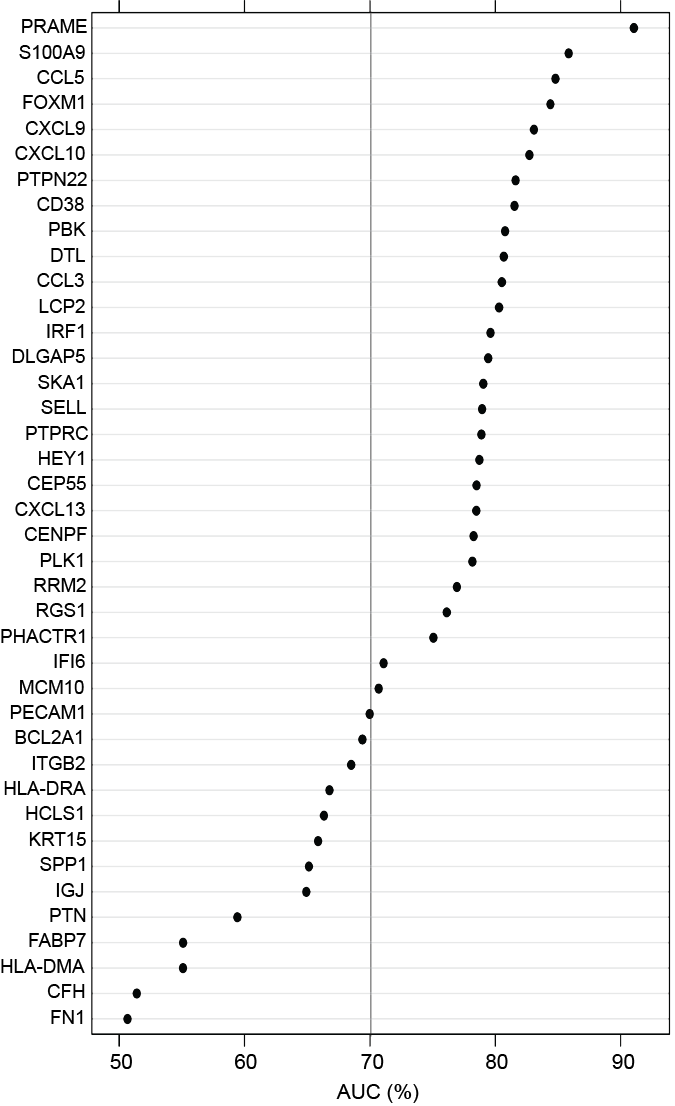

Supplement: Supplementary file 2 — Fig. S1. Performance of the 40 genes tested in the training cohort. The performance of each gene is measured by the area under the curve (AUC) of each gene when differentiating benign and malignant melanocytic lesions. [file CUP-42-244-s001.doc]
