# Supplementary material for: Clinical validation of a gene expression signature that differentiates benign nevi from malignant melanoma
Source: J Cutan Pathol. 2015 Apr 13;42(4):244–52. doi: 10.1111/cup.12475 (PMC6681167; doi:10.1111/cup.12475)

**Supplemental Figure 2. Clustering of the gene expression of the 40 genes evaluated in the training cohort.** The three main clusters are annotated. CCP=cell cycle progression.


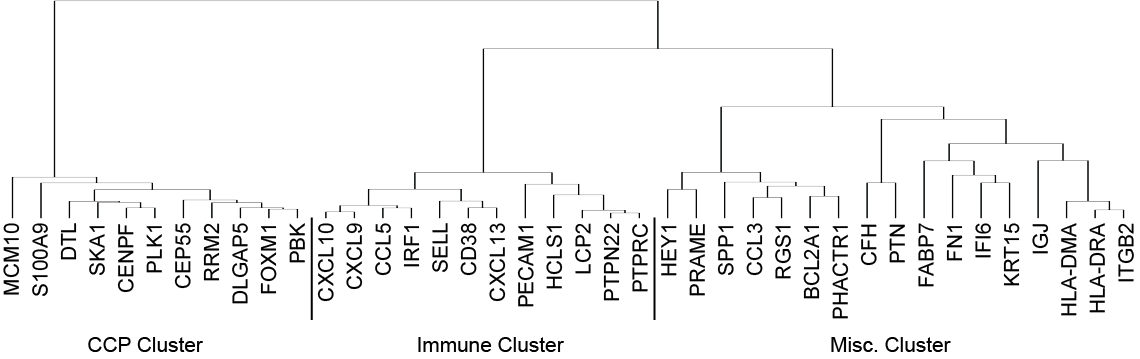

Supplement: Supplementary file 3 — Fig. S2. Clustering of the gene expression of the 40 genes evaluated in the training cohort. The three main clusters are annotated. CCP, cell cycle progression. [file CUP-42-244-s006.doc]
