# Supplementary material for: Clinical validation of a gene expression signature that differentiates benign nevi from malignant melanoma
Source: J Cutan Pathol. 2015 Apr 13;42(4):244–52. doi: 10.1111/cup.12475 (PMC6681167; doi:10.1111/cup.12475)

**Supplemental Figure 3. Performance of the best multivariate model in the training cohort.** A) Distribution of the diagnostic score from the model in malignant and benign samples. B) The ROC curve of the model, with the sensitivity and specificity displayed at the chosen cutoff point. The AUC of the ROC is also noted.


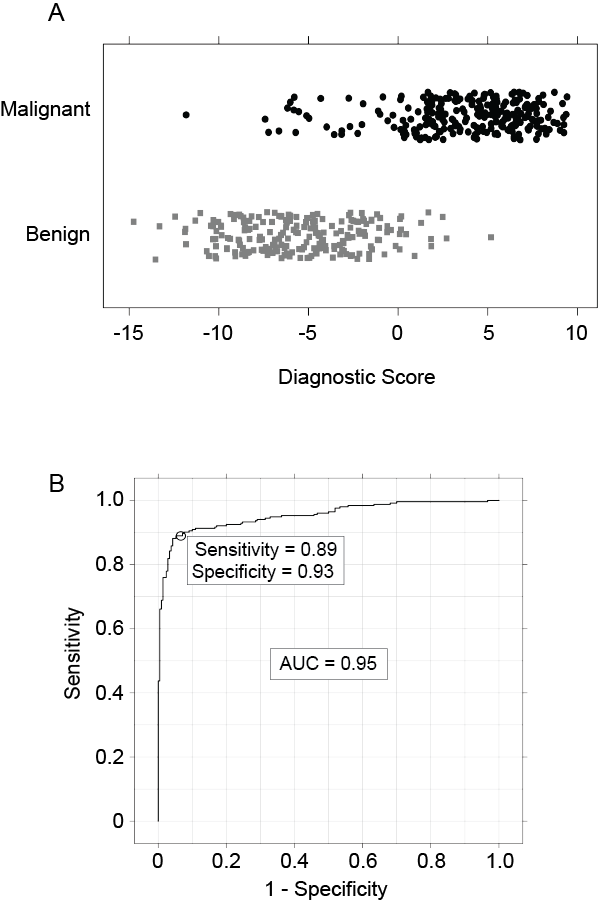

Supplement: Supplementary file 4 — Fig. S3. Performance of the best multivariate model in the training cohort. A) Distribution of the diagnostic score from the model in malignant and benign samples. B) The receiver operating characteristic (ROC) curve of the model, with the sensitivity and specificity displayed at the chosen cutoff point. The area under the curve (AUC) of the ROC is also noted. [file CUP-42-244-s004.doc]
