# Supplementary material for: Clinical validation of a gene expression signature that differentiates benign nevi from malignant melanoma
Source: J Cutan Pathol. 2015 Apr 13;42(4):244–52. doi: 10.1111/cup.12475 (PMC6681167; doi:10.1111/cup.12475)
Supplement: Supplementary file 5 — Table S1. List of the 79 candidate biomarker genes. [file CUP-42-244-s003.doc]

**Supplemental Table 1. List of the 79 candidate biomarker genes**

| *ARPC2* | *CXCL12* | *IRF1* | *PRC1* |
| --- | --- | --- | --- |
| *ASF1B* | *CXCL13* | *IRF4* | *PTN* |
| *ASPM* | *CXCL9* | *ITGB2* | *PTPN22* |
| *BCL2A1* | *DLGAP5* | *KIAA0101* | *PTPRC* |
| *BIRC5* | *DTL* | *KIF11* | *PTTG1* |
| *BUB1B* | *FABP7* | *KIF20A* | *RAD51* |
| *CCL19* | *FN1* | *KRT15* | *RAD54L* |
| *CCL3* | *FOXM1* | *LCP2* | *RGS1* |
| *CCL5* | *GDF15* | *MCM10* | *RRM2* |
| *CD38* | *HCLS1* | *NCOA3* | *S100A9* |
| *CDC20* | *HEY1* | *NR4A1* | *SELL* |
| *CDCA3* | *HLA-DMA* | *NUSAP1* | *SERPPINB4* |
| *CDCA8* | *HLA-DPA1* | *ORC6L* | *SKA1* |
| *CDK1* | *HLA-DPB1* | *PBK* | *SOCS3* |
| *CDKN3* | *HLA-DRA* | *PECAM1* | *SPP1* |
| *CENPF* | *HLA-E* | *PHACTR1* | *TK1* |
| *CENPM* | *IFI6* | *PHIP* | *TOP2A* |
| *CEP55* | *IGHM* | *PLK1* | *WIF1* |
| *CFH* | *IGJ* | *POU5F1* | *WNT2* |
| *CXCL10* | *IGLL5;CKAP2* | *PRAME* |  |
