# Supplementary material for: Clinical validation of a gene expression signature that differentiates benign nevi from malignant melanoma
Source: J Cutan Pathol. 2015 Apr 13;42(4):244–52. doi: 10.1111/cup.12475 (PMC6681167; doi:10.1111/cup.12475)
Supplement: Supplementary file 6 — Table S2. Performance of the candidate diagnostic genes in a multivariate model using the training cohort. [file CUP-42-244-s002.doc]

**Supplemental Table 2. Performance of the candidate diagnostic genes in a multivariate model using the training cohort**.

| **Gene** | **Function** | ***p*-value in multivariate model** |
| --- | --- | --- |
| *CENPF* | Cell Cycle Progression | NS* |
| *CEP55* | Cell Cycle Progression | NS* |
| *DLGAP5* | Cell Cycle Progression | NS* |
| *DTL* | Cell Cycle Progression | NS* |
| *FOXM1* | Cell Cycle Progression | NS* |
| *MCM10* | Cell Cycle Progression | NS* |
| *PBK* | Cell Cycle Progression | NS* |
| *PLK1* | Cell Cycle Progression | NS* |
| *RRM2* | Cell Cycle Progression | NS* |
| *SKA1* | Cell Cycle Progression | NS* |
| *PRAME* | Cell Differentiation | 4.5 x 10^-28^ |
| *PTN* | Cell Differentiation | NS |
| *FABP7* | Fat Metabolism | NS |
| *S100A9* | Immune | 3.9 x 10^-12^ |
| *CCL5* | Immune | 7.2 x 10^-5 †^ |
| *CD38* | Immune | 7.2 x 10^-5 †^ |
| *CXCL10* | Immune | 7.2 x 10^-5 †^ |
| *CXCL9* | Immune | 7.2 x 10^-5 †^ |
| *IRF1* | Immune | 7.2 x 10^-5 †^ |
| *LCP2* | Immune | 7.2 x 10^-5 †^ |
| *PTPRC* | Immune | 7.2 x 10^-5 †^ |
| *SELL* | Immune | 7.2 x 10^-5 †^ |
| *BCL2A1* | Immune | NS |
| *CCL3* | Immune | NS |
| *CFH* | Immune | NS |
| *CXCL13* | Immune | NS |
| *HCLS1* | Immune | NS |
| *HLA-DMA* | Immune | NS |
| *HLA-DRA* | Immune | NS |
| *IFI6* | Immune | NS |
| *IGJ* | Immune | NS |
| *ITGB2* | Immune | NS |
| *PECAM1* | Immune | NS |
| *PTPN22* | Immune | NS |
| *RGS1* | Immune | NS |
| *SPP1* | Immune | NS |
| *FN1* | Signaling | NS |
| *HEY1* | Signaling | NS |
| *KRT15* | Structural | NS |
| *PHACTR1* | Structural | NS |

*p-*values are Bonferonni adjusted.

NS, not significant (*p*-value >0.05).

* All 10 cell cycle progression genes were evaluated as an averaged group in the multivariate model.

† These eight immune genes were evaluated as averaged group in the multivariate model.
